# Comparison of carpal tunnel release with double mini-incision approach and traditional approach: A retrospective study

**DOI:** 10.1097/MD.0000000000042510

**Published:** 2025-05-16

**Authors:** Chenfei Li, Yuxin Liu, Bing Zhang, Jian Lu, Xuyang Shi, Yipeng Yang, Lingde Kong

**Affiliations:** a Hebei Medical University Third Hospital, Shijiazhuang, Hebei, PR China; b The Fourth Hospital of Hebei Medical University, Shijiazhuang, Hebei, PR China.

**Keywords:** carpal tunnel syndrome, double mini-incision, pillar pain, surgical details, traditional approach, wound pain

## Abstract

The aim of this study was to investigate the safety and efficacy of the double mini-incision approach, and to clarify its surgical details. We retrospectively enrolled 82 patients with primary carpal tunnel syndrome. Among them, 30 patients with conventional approach were enrolled in group A, and the other 52 patients with double mini-incision approach were enrolled in group B. Objective tests were performed on patients, and basic information and subjective evaluation of patients were collected. The surgical effects and complications of the 2 approaches were compared. In addition, the surgical details of double mini-incision were further explored. The incision length of group B (26.1 ± 6.1 mm) was significantly shorter than that of group A (45.7 ± 5.9 mm, *P* ＜ .001). Patients in group B (93.7 ± 5.4) had significantly higher satisfaction with incision appearance than those in group A (84.3 ± 6.1, *P* ＜ .001). At the 12-month follow-up, no statistically significant difference in clinical outcomes were observed between the 2 groups (*P* > .05). However, there were 2 cases with wound pain and 1 case with pillar pain in group A, but none in group B. Two patients in group B who underwent the distal incision 1st were transferred to the conventional approach because of the epineurium and perineurium injury. The double mini-incision approach offers a sufficient range of release and surgical field, resulting in favorable surgical outcomes. The proximal incision made 1st helps to reduce the risk of nerve injury.

## 1. Introduction

Carpal tunnel syndrome (CTS) is the most common peripheral nerve entrapment syndrome in clinical practice, which is highly related to frequent wrist movements at work.^[1]^ The symptoms of CTS are mainly paresthesia, numbness, tingling and pain in the skin innervated by the median nerve.^[2]^ As the disease progresses, the muscles on the radial side of the hand will appear disuse weakness and atrophy.^[3]^ Once CTS progresses to the middle and late stages, surgical release of carpal tunnel is the only and effective way.^[4]^

The traditional surgical approach is a longitudinal incision from the palm to the wrist, which provides a good surgical field and can reliably release the transverse carpal ligament.^[5]^ However, there is a growing body of research that links traditional approaches to persistent complications such as scar tenderness and pillar pain.^[6]^ To reduce these complications, a variety of minimally invasive approaches have gradually emerged. Yong-Suk Lee et al performed the procedure through a single small transverse incision at the wrist crease and achieved satisfactory results.^[7]^ Saran Malisorn reported a single longitudinal small palmaris incision for the treatment of CTS that successfully reduced the occurrence of pain-related complications.^[8]^ However, a single small incision often requires special surgical instruments, such as hook knife or protective guide plate, to achieve full release.^[7–9]^ It will take some time for these special devices to become widespread. In addition, for severe CTS, the ability of a single small incision to deal with the deep fascia of the distal forearm and the thickened interthenar aponeurosis remains unclear.^[10]^ A single small incision may also not be able to cope with the structural variation that arises in the carpal tunnel.^[11]^

After reviewing previous literature, we think double mini-incision still has its unique advantages for the treatment of CTS. In order to further evaluate its safety and efficacy, this study compare it with the conventional approach. Additionally, we tried to provide a comprehensive understanding of the surgical details involved in the double mini-incision technique.

## 2. Materials and methods

### 2.1. Participants

The protocol for the study was approved by the Ethics Committee of Hebei Medical University Third Hospital (No. 2024-028-1), and all investigations were conducted in conformity with ethical principles. This study met the conditions for exemption of informed consent and was approved by the Ethics Committee of the Third Hospital of Hebei Medical University. The data used in this study was anonymized before its use. This work has been reported in line with the STROCSS (Strengthening the Reporting of Cohort Studies in Surgery) criteria.^[12]^ We included consecutive patients diagnosed with primary CTS who had carpal tunnel release from March 2021 to December 2022. Before January 2022, the conventional approach was used to release carpal tunnel, and after that, the double mini-incision approach was applied as a new method. The diagnosis was made based on the patient’s symptoms and signs, including median nerve sensory abnormalities, dysesthesia, night pains, thenar atrophy, dysfunction of thumb opposition, positive Tinel’s test, and positive carpal tunnel pressure test. Finally, B-scan ultrasonography and electromyography were used to determine the median neuropathy in the wrist and to exclude other diseases.

Exclusion criteria were presence of bilateral symptomatic CTS, inflammatory joint disease, gout, a combined nerve compression, previous hand or upper extremity surgery, conservative treatment with steroid injections, and incomplete follow-up data.

### 2.2. Surgical procedure

The patient was placed in a supine position on the operating table, and the affected hand, wrist, and forearm were cleaned with povidone-iodine solution. Anesthesia was initiated through local infiltration using 2 mL of lidocaine solution with a concentration of 0.1%. Upper arm inflatable tourniquet inflated at a range of 240 to 280 mm Hg.

For the conventional approach, a “S” shaped incision was made from the proximal part of the thenar striae to the ulnar side of the wrist striae. After the skin and subcutaneous tissue were dissected, the transverse carpal ligament was cut to expose the median nerve. If severe epineurial fibrosis is detected during the surgical procedure, we would perform release epineurotom. The tourniquet was released, and the wound was routinely sutured after compression hemostasis.

**Figure 1.**
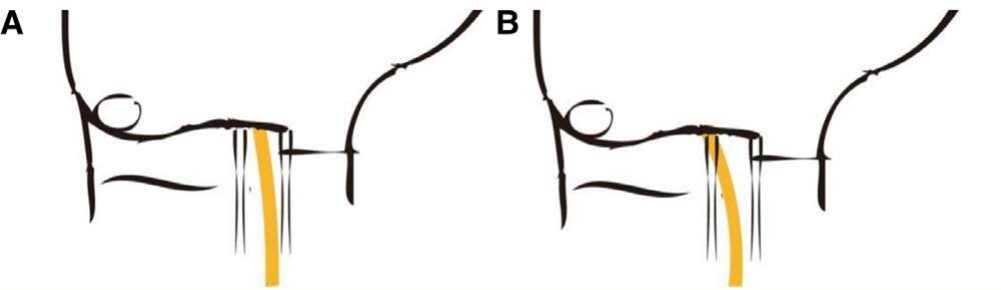
Anatomical variations of the median nerve at wrist. (A) Normal median nerve anatomy. (B) Structural variation of median nerve located on the ulnar side of palmaris longus.

**Figure 2.**
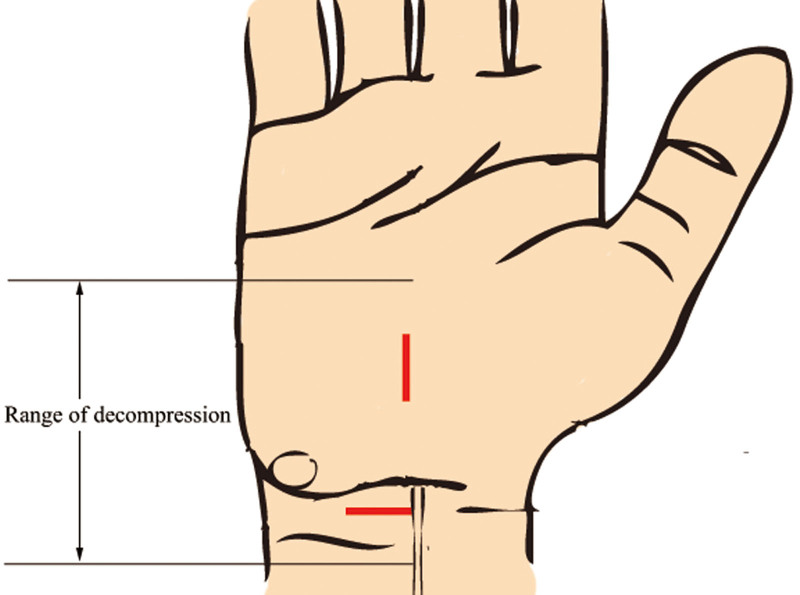
Effective release range of double mini-incision approach.

**Figure 3.**
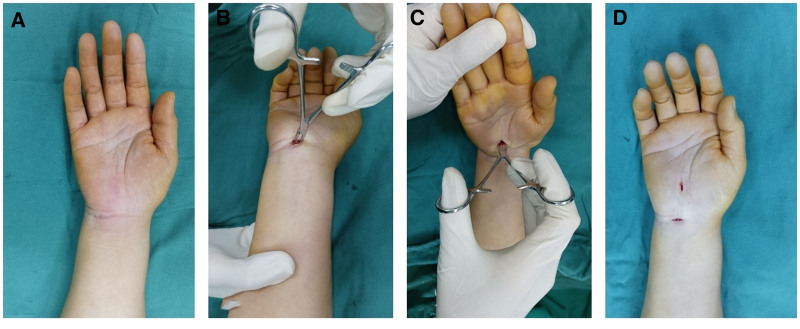
Surgical diagram of the double mini-incision approach. (A) Preoperative diagram. (B) Carpal tunnel release was performed through the proximal incision under dorsiflexion of the wrist. (C) Carpal tunnel release was performed through the distal incision under volar flexion of the wrist. (D) Double mini-incision diagram.

For the double mini-incision approach, the proximal incision is located at the wrist crease starting from the ulnar side of the palmaris longus. After subcutaneous adipose tissue was bluntly dissected and volar carpal ligament was cut, median nerve may show or be on the radial side of the visual field (Fig. 1). The distal incision starts at 0.5 cm distal to the highest point of the eminence between thenar and hypothenar on the radial border of the ring finger line. Subcutaneous adipose tissue and palmaris aponeurosis were bluntly dissected using vascular forceps until the transverse carpal ligament was exposed. The length of both incisions ranged from 1 to 1.5 cm. The proximal incision is used to release the deep fascia of the distal forearm and the proximal part of the transverse ligament of the wrist which included the inlet. The distal incision is used to release the thickened interthenar aponeurosis and the distal part of the transverse ligament of the wrist which included the outlet. The entire transverse ligament of the wrist was completely incised and release was completed (Fig. 2). The combination of different wrist positions can provide a enough surgical field. Wrist dorsiflexion helps to release the carpal tunnel inlet in the proximal incision, and wrist volar flexion helps to release the carpal tunnel outlet in the distal incision (Fig. 3). Finally, the wound was routinely sutured and bandaged.

All procedures were performed by the same surgeon. No special surgical instruments and splints were used in the 2 groups. Methylcobalamin tablets were given orally at 0.5 mg 3 times a day for 1 month after surgery. Finger movement was started on the 2nd day after surgery, and the stitches were removed 2 weeks after surgery.

### 2.3. Outcome evaluation

Basic information of patients was collected before surgery, including age, gender, body mass index, affected side and duration of symptoms. Corresponding objective tests were performed, grip and pinch strength were assessed with an E-LINK electronic gripping power device and electronic pinch strength device. Each measurement was performed twice, with an interval of 10 minutes, and the average value was calculated. The 2-point discrimination test was conducted with the patient’s eyes closed, and 2 sharp points were used to measure the index finger. In addition, a series of subjective evaluations were completed. Wrist pain was evaluated using the visual analogue scale, with a score of 0 indicating no pain and a score of 10 indicating maximum pain. The Levine score is divided into symptom and functional components, with a score of 1 being the mildest and a score of 5 being the most severe on both scales.^[13]^ Disabilities of the arm, shoulder and hand were used to evaluate patients’ ability to perform daily activities, with score ranging from 0 to 100.^[14]^ Incision length and operation time were recorded during operation. At the 1st dressing change after surgery, patients rated the aesthetics of the incision, with 100 representing the most satisfied and 1 representing the least satisfied.

Patients were followed up at 2 weeks, 1 month, 3 months, 6 months and 12 months after surgery. Wound pain was considered when located centrally underneath the surgery scar. Pillar pain was defined as discomfort at the thenar or hypothenar eminence or both while tightly gripping the hand. Besides, all patients performed every objective tests and subjective evaluations again at the final follow-up.

### 2.4. Statistical analyses

We performed all statistical analyses using the Statistical Package for Social Sciences 26.0 (IBM Corporation, Armonk, New York). In descriptive analysis, means and standard deviations were used for continuous variables and frequencies as well as percentages were used for categorical variables. To determine the difference between groups, Fisher exact tests or independent-samples *t* tests were used. *P* < .05 was considered to indicate a statistically significant difference.

## 3. Results

A total of 82 patients with CTS were included in this study. There were 30 patients with conventional approaches included in group A, and 52 patients with double mini-incision approaches included in group B. The mean age of the patients in group A were 53.9 ± 9.7 years and that in group B were 52.8 ± 10.1 years. The duration of symptoms in the 2 groups were 6.7 ± 3.8 months and 6.3 ± 3.4 months respectively. There were no iatrogenic vascular or tendon injuries during the surgery in either group. No instances of wound infection or recurrent symptoms were observed among the patients. There were no significant differences in baseline, pinch strength, grip strength, 2-PD, VSA, Levine score, and DASH score between the 2 groups (Table 1).

**Table 1:** Baseline characteristics of patients with conventional approach (group A) and double mini-incision approach (group B).

	Group A	Group B	*P* value
Number of patients	30	52	
Age (yr)	53.9 ± 9.7	52.8 ± 10.1	.630
Gender (male/female)	19/11	30/22	.648
BMI (kg/m^2^)	23.8 ± 4.9	24.7 ± 4.5	.396
Affected side (left/right)	13/17	24/28	.822
Duration of symptoms (mo)	6.7 ± 3.8	6.3 ± 3.4	.621
Pinch strength (g/mm^2^)	4.3 ± 1.7	4.5 ± 1.9	.632
Grip strength (g/mm^2^)	16.8 ± 7.9	16.5 ± 8.4	.873
2-Point discrimination (mm)	7.3 ± 1.8	7.0 ± 1.5	.570
VAS score	4.3 ± 1.2	3.9 ± 1.5	.206
Levine symptom score	2.9 ± 0.5	2.8 ± 0.7	.331
Levine function score	2.5 ± 0.4	2.3 ± 0.4	.210
DASH score	32.9 ± 17.6	34.1 ± 18.4	.773

BMI = body mass index, DASH = disabilities of the arm, shoulder and hand, VAS = visual analogue scale.

Details of the operation and follow-up results are provided in Table 2. There were no difference in operative time between the 2 groups (*P* = .312), while the incision length was significantly shorter in group B (26.1 ± 6.1 mm) than in group A (45.7 ± 5.9 mm, *P* ＜ .001). Patients in group B (93.7 ± 5.4) had significantly higher satisfaction with incision appearance than those in group A (84.3 ± 6.1, *P* ＜ .001). At the 12-month follow-up, no statistically significant difference in clinical outcomes were observed between the 2 groups (*P* > .05). Wrist pain completely disappeared or significantly relieved in all patients. However, there were 2 cases of wound pain and 1 case of pillar pain in group A, but none in group B.

**Table 2:** Comparison of outcomes in patients with conventional approach (group A) and double mini-incision approach (group B).

	Group A	Group B	*P* value
Operation time (min)	24.2 ± 5.0	25.3 ± 4.6	.312
Incision length (mm)	45.7 ± 5.9	26.1 ± 6.1	＜.001
Appearance score	84.3 ± 6.1	93.7 ± 5.4	＜.001
Pinch strength (g/mm^2^)	6.7 ± 2.2	6.5 ± 2.4	.698
Grip strength (g/mm^2^)	24.8 ± 6.3	24.4 ± 6.0	.829
2-Point discrimination (mm)	3.1 ± 1.0	3.2 ± 0.9	.632
VAS score	0.5 ± 0.4	0.5 ± 0.3	.442
Levine symptom score	1.4 ± 0.5	1.5 ± 0.4	.502
Levine function score	1.2 ± 0.4	1.3 ± 0.4	.231
DASH score	10.5 ± 7.8	9.9 ± 6.3	.656
Wound pain (yes/no)	2/28	0/52	.131
Pillar pain (yes/no)	1/29	0/52	.366

DASH = disabilities of the arm, shoulder and hand, VAS = visual analogue scale.

The median nerve of 11 (21%) patients could be directly observed in the proximal incision, and the median nerve of the other 41 (79%) patients was located in the radial of the palmaris longus. Group B was further divided into 2 subgroups according to the sequence of double mini-incisions in the surgical records (Table 3). Patients with proximal incision 1st were included in group B1, and those with distal incision 1st were included in group B2. In group B1, after the proximal incision was released, it was found that there were still 17 patients (68%) who were not released completely after the distal incision was made. In group B2, after the distal incision was released, it was found that there were still 17 cases (63%) who were not released completely after the proximal incision was made. In the patients who made distal incision 1st, 2 patients experienced abnormal pain and release procedure ceased immediately. After switching to the conventional approach, epineurium and perineurium injuries of median nerve were found. These 2 cases showed no motion abnormality and have good recovery during follow-up, but were not included in statistical analyses.

**Table 3:** Comparison of outcomes in patients with cut proximal incision 1st (group B1) and cut distal incision 1st (group B2).

	Group B1	Group B2	*P* value
Number of patients	25	27	
Operation time (min)	25.5 ± 4.4	25.2 ± 4.8	.834
Incision length (mm)	25.4 ± 6.1	26.9 ± 6.0	.374
Appearance score	93.0 ± 5.3	94.2 ± 5.4	.948
Pinch strength (g/mm^2^)	6.6 ± 2.5	6.3 ± 2.3	.662
Grip strength (g/mm^2^)	25.0 ± 5.7	24.0 ± 6.2	.531
2-Point discrimination (mm)	3.0 ± 0.8	3.4 ± 0.9	.127
VAS score	0.5 ± 0.2	0.5 ± 0.3	.645
Levine symptom score	1.6 ± 0.4	1.5 ± 0.3	.321
Levine function score	1.3 ± 0.3	1.4 ± 0.4	.330
DASH score	10.3 ± 6.1	9.5 ± 6.5	.644

DASH = disabilities of the arm, shoulder and hand, VAS = visual analogue scale.

## 4. Discussion

The release of the transverse carpal ligament can be achieved through a variety of surgical approaches. This study compared the double mini-incision approach with the conventional approach, and it was found that the use of double mini-incision may effectively reduced postoperative complications and improved patient satisfaction. Besides, the results showed that making the proximal incision 1st could better cope with the occurrence of structural variation in the carpal tunnel, and minimized risks of nerve injury during surgery.

The traditional “S” approach is almost the “gold standard” in carpal tunnel release and has a definite curative effect.^[8]^ In our study, both the conventional approach and the double mini-incision approach effectively treated CTS, and all patients experienced relief of neurological symptoms. However, Abdullah AF et al reported that 24% of postoperative complications in CTS were attributed to the length of the incision.^[15]^ The double mini-incision reduces the incision length of the conventional approach by nearly half and avoids the main load bearing area. This averts scar tenderness when the patient performs activities such as mouse operation in daily life and reduces the incidence of wound pain and pillar pain. Moreover, this approach has a similar effective release range as the conventional approach, and can provide more satisfactory incision appearance. At the same time, the double mini-incision approach does not increase the cost of patients and the operation time. Therefore, we believe that the double mini-incision approach is superior to the traditional approach for carpal tunnel release.

Various limited incision approaches have been applied to CTS to reduce incision-related complications.^[16]^ In general, these studies have used novel surgical instruments to achieve effective release or provide median nerve protection within a limited incision.^[17–19]^ It takes a long process for new surgical instruments to be developed, marketed, and popularized in clinical practice. In addition, the effects of a single small incision on the deep fascia of the distal forearm and thickened interthenar aponeurosis are uncertain.^[20]^ This was also confirmed by the fact that more than half of the patients in this study were unable to achieve effective carpal tunnel release through a single incision. Although arthroscopy can provide a direct examination of the wrist transverse ligament and median nerve, this technique is more complex and carries a relatively higher economic burden for patients.^[21]^ Compared with the single mini-incision and arthroscopic, the double mini-incision increased the incision length by about 1 cm, but it provided a full visible surgical field and obtained a larger decompression area. Besides, the double mini-incision technique has relatively low technical difficulty and short learning curve, which is more suitable for primary medical institutions.

Although the double mini-incision approach was 1st proposed at the year of 1993, many surgical details has not been clarified.^[22]^ The incisions avoid the weight-bearing area at the root of the palm, but there is inconsistency in the description of the distal incision.^[22–25]^ The differences focused on whether the distal incision was near the center of the palm or not, and most choose to cut distally from the intersection of Kaplan cardinal line and radial side ring finger. Different from there study, the distal incision in our study was 0.5 cm distal to the eminence of thenar and hypothenar, which is the arc-shaped descent zone distal to the root of the palm. Firstly, in daily life, whether holding or lifting activities will be more contact with the skin of the center of the palm. Secondly, the release of each incision in the double mini-incision approach is bidirectional, and the entire length of the median nerve in the wrist can be seen through the incision. Therefore, we believe that the location of the distal incision is not necessary to reach the center of the palm.

There were 2 cases of epineurium and perineurium injury in patients who had a distal incision 1st in our study. Anatomical variation in the median nerve were observed after switching to the conventional approach. The normal median nerve enters the carpal tunnel from the radial side of the palmaris longus, whereas in these 2 cases the median nerve is located on the ulnar side of the palmaris longus. This variation was reported by Russell Payne et al, who dissected 76 wrist specimens, of which 24 (32%) had the median nerve located on the ulnar side of the wrist.^[26]^ Therefore, during the operation, we should 1st observe the path of the median nerve through the proximal incision, and then perform the distal release. There are other variations in the median nerve in the carpal tunnel, but none were observed in this study. High bifurcation of median nerve, another common variation in which the median nerve bifurcates at the proximal end of the transverse carpal ligament, occurs in about 1% to 3.3% of CTS patients.^[27]^ A rare variation has been reported where the motor branch takes off from an ulnar and anterior location, bridging the median nerve as it approaches the thenar musculature.^[27,28]^ Each of these variants has the potential to cause nerve damage when the distal incision is performed first. The proximal incision can identify the variation of the median nerve, which is one of the key and advantages of double mini-incision for carpal tunnel release.

There are still several limitations of our study. First, this study is retrospective study, and there was no statistical difference in complication between the 2 approaches, which may be due to insufficient sample size. Second, only the double mini-incision approach and the conventional approach were compared in this study. Whether the double mini-incision approach still has advantages over other incisions or arthroscopy needs further study. Finally, median nerve conduction velocity measured by electromyography during follow-up may more accurately evaluate the prognosis.

## 5. Conclusion

In summary, the double mini-incision approach offers a sufficient range of release and surgical field, resulting in favorable surgical outcomes, reduced complications, and improved aesthetic appearance. The proximal incision can better observe the variation of the median nerve in the carpal tunnel. Therefore, the proximal incision made 1st helps to reduce the risk of nerve injury.

## Author contributions

**Conceptualization:** Chenfei Li, Lingde Kong.

**Data curation:** Yuxin Liu, Bing Zhang, Jian Lu.

**Formal analysis:** Yuxin Liu.

**Investigation:** Bing Zhang, Xuyang Shi, Yipeng Yang.

**Software:** Jian Lu.

**Writing – original draft:** Chenfei Li.

**Writing – review & editing:** Yuxin Liu, Xuyang Shi, Yipeng Yang, Lingde Kong.
